# Tuberculosis in the middle of COVID-19 in Morocco: efforts, challenges and recommendations

**DOI:** 10.1186/s41182-021-00388-y

**Published:** 2021-12-20

**Authors:** Oumnia Bouaddi, Mohammad Mehedi Hasan, Abdul Moiz Sahito, Pritik A. Shah, Abdelrahman Zaki Ali Mohammed, Mohammad Yasir Essar

**Affiliations:** 1 Faculty of Dentistry, Mohammed V University, Rabat, Morocco; 2 Department of Biochemistry and Molecular Biology, Faculty of Life Science, Mawlana Bhashani Science and Technology University, Tangail, Bangladesh; 3 Division of Infectious Diseases, The Red-Green Research Centre, BICCB, Dhaka, Bangladesh; 4 Dow University of Health Sciences, Karachi, Pakistan; 5 Bangalore Medical College and Research Institute, Bangalore, India; 6 Misr University for Science and Technology, Giza, Egypt; 7 Kabul University of Medical Sciences, 1001 Kabul, Afghanistan

**Keywords:** TB, Tuberculosis control, COVID-19, Public health, Morocco

## Abstract

Tuberculosis (TB) is a deadly infectious disease that kills approximately 1.5 million people per year and is among the most frequent respiratory infections in developing countries. Morocco has made significant progress in the control and management of TB during the past 30 years thanks to its National Plan for Tuberculosis and the continuous support of national and international partners. While tremendous efforts were undertaken to tilt the balance against the COVID-19 pandemic, new challenges resurfaced with regard to long-standing health problems amongst which is TB. The spill-over effect of the COVID-19 pandemic disrupted health service delivery globally, threatening to reverse years of progress made on the TB control front. In Morocco, this crisis highlighted deep shortcomings within the national health system and in the adopted approach to TB control. This article discusses national efforts to get back on track with regard to TB management, the multitude of challenges that co-emerged with the onset of COVID-19 and lays down key recommendations to implement in order to build back a TB control plan that is resilient in the face of health hazards.

## Introduction

It has been more than three decades since Morocco put in place a national Tuberculosis (TB) control program. Morocco was also amongst the first nations to have adopted the DOTS (Directly Observed Treatment Short-course) program in the early 1990s [1]. It is estimated that the Moroccan National Tuberculosis Program (NTP) has surpassed international goals and standards for case identification and successful treatment rates over the past decade [1]. The Moroccan government has provided and supported the NTP since its culmination and offers free services, advice and treatment to patients.

As of 2019, the incidence of TB in Morocco was 97 cases for each 100,000 inhabitants, compared to 99 cases per 100,000 inhabitants in the previous year, which means a decrease of only 2.02% [2]. In Morocco, the spatial distribution of TB is not accidental. A sustained spatial clustering of high TB incidence rates has been observed in the northwestern part of the country compared to the southern and eastern parts [3].

New challenges emerged as a result of the spillover impact of the pandemic, which disrupted many global health systems [4–6]. The Moroccan population suffered a great deal during these times. Mobility restrictions and physical distancing measures threatened to undo years of advances made on the control front. This is evidenced by an alarming decrease in the recommended uptake of vaccines due to the growing reluctance of parents and the postponement of appointments in public and private health facilities. The direct and indirect effects played a role in harming the buildings of progress in public health. One such example is the TB program and its patient population. Because of the crippling attributes of COVID-19 national healthcare systems, numerous nations have experienced TB eradication blockades [7–9]. By extrapolating the cause–effect relationships during the COVID-19 pandemic on Morocco's struggle against TB, it is clear that the situation is fairly comparable.

This article aims to examine the current situation of TB in Morocco with a view to understanding the current efforts and challenges, and identifying opportunities for improvement through some key recommendations.

## Ongoing efforts and current challenges in TB management during the COVID-19 pandemic in Morocco

Morocco has a total population of 36,910,558 (2020) with an average life expectancy of 76 years (2019). As a country that has recently underwent an epidemiological transition, the main causes of death are—among many other non-communicable diseases—stroke and ischemic heart disease. However, TB remains the most common infectious cause of death after neonatal disorders and low respiratory infections [10].

The National Tuberculosis Prevention and Control Plan has been in effect for over three decades and is a top priority in the country's healthcare system. With substantial support from the Global Fund to fight Malaria, Tuberculosis, and AIDS, Morocco was able to achieve an 80% reduction in the number of TB primary infections between 1980 and 2018. Therapeutic success rate also reached 85% in 1995 and has remained above 86% since 2002, surpassing the global average. In 1991, Morocco introduced the DOTS strategy in line with WHO recommendations, which allowed the country to provide free of charge standardized TB treatment regimen, strengthen its primary healthcare structures in case detection, treatment and patient follow-up, secure a regular, uninterrupted supply of all essential anti-TB drugs and increase laboratory capacity. In 2006, the country also joined STOP TB Strategy to consolidate its previous efforts and design new interventions that aim to address drug-resistant TB and co-infections with HIV. The 2006–2015 plan resulted in a 59% decrease in TB-related mortalities and a 27% decrease in TB prevalence, thereby achieving target 6C of the Millennium Development Goals [11, 12].

In 2019, 179.4 deaths attributed to multi-drug resistant TB (MDR-TB)—without extensive drug resistance (XDR)—were recorded. The death rate amongst females was twice as high compared to males for both drug susceptible and MDR-TB. Only 11% of new TB cases were tested for MDR-TB in 2019. However, in 2020, 50% of bacteriologically confirmed TB infections were screened for rifampicin resistance (RR), 18 402 cases of MDR/RR-TB were identified and 17 678 were started on treatment [13].

In 2020, the country recorded 29,018 new cases of TB all of which were put under treatment, thereby almost achieving its STOP TB target of 30,100 cases diagnosed and treated [14]. Furthermore, on World Tuberculosis Day 2020, the Ministry of Health (MoH) in Morocco announced an extension of the NTP with the aim of attaining a 60% reduction of TB-related deaths in 2023 compared to 2015. The MoH also called for intensifying the fight against TB by consolidating national and international efforts, in a manner analogous to the convergence in public policies and the multi-sectoral collaboration observed during the COVID-19 response [15]. Additionally, The Global Fund for AIDS, TB and Malaria has earmarked 30 million Moroccan Dirhams to support the country’s endeavors during the period 2021–2023.

Although the efforts undertaken managed to reverse the trend in TB mortality and infection, the annual decrease of incidence—while regular—remains as low as 1,1% which forestalls the prospect of “Ending Tuberculosis by 2030”. This challenge is likely due to the epidemiological affinity of TB to high-density regions evidenced by a staggering 86% of TB cases recorded in marginal neighborhoods in 6 regions where 79% of the Moroccan population lives. Worthy of note are the socio-economic conditions that underlie the disease such as poverty, unemployment, malnutrition, and inadequate housing which exert a powerful influence and compromise the desired health outcomes of TB programs and interventions.

Along with constrained financial resources, healthcare workforce shortage and an insufficient laboratory capacity, new challenges emerged due to the indirect effects of the COVID-19 crisis that disrupted global health systems. Morocco had initially succeeded in controlling the spread of SARS-CoV-2 by imposing a country-wide lockdown and suspending incoming flights. However, the resulting economic downturn led to a subsequent easing of these measures in June 2020. As of December 2021, Morocco is still in a state of emergency, but has lifted all movement restrictions and night curfews with the exception of mass gatherings. The Moroccan authorities also made the vaccine pass mandatory for work, travel and access to public places. This decision was met with criticism, resistance and even protests in the streets led by citizens and politicians. With the emergence of the new Omicron variant, a swift closing of the Moroccan borders has also occurred.

The restrictions on mobility and physical distancing policies may have been effective in containing the COVID-19 outbreak, but they have also threatened to reverse years of progress made on the TB control front. This is evidenced by an alarming decline in the uptake of recommended vaccines due to the growing hesitancy amongst parents and the postponement of appointments in both public and private healthcare facilities. According to a cross-sectional study conducted in 12 Moroccan cities, only 26% of physicians declared having used Ministry guidelines to inform their decisions regarding child immunization during COVID-19 [16]. Furthermore, the country has witnessed a 6% reduction in TB case notifications in 2020 compared to 2019 [14].

Another major barrier to optimal TB care is patient adherence to treatment and loss to follow-up, which are further undermined by COVID-19 restrictive measures. This challenge could be remedied through effective communication between healthcare providers and their patients about the disease, the duration of treatment and the consequences of not completing it [17]. Efforts were undertaken by non-profit organizations to integrate traditional TB management with a patient-centered method by deploying mobile technology, Medication Event Monitoring System and Smart pillboxes to increase patient compliance in hard-reached regions where the burden is high, as well as improve TB case management [18, 19]. However, these approaches require leveraging well-designed community health worker programs which are yet to be developed and integrated into the formal Moroccan health system.

The emerging COVID-19 variants of concern (VoC) also threaten to put additional stress on Morocco’s resources in terms of workforce and budget allocation, while the country is just starting to recover from the pandemic-induced economic downturn.

## Recommendations

The COVID-19 pandemic has emphasized that guaranteeing uninterrupted access to essential services and operations that address long-standing health problems, is the key to preserving the lives of individuals living with TB and other related health conditions. Safeguarding or improving TB control during health emergencies therefore requires consistent and innovative practices such as:

1. Leveraging the newly introduced telemedicine for remote consultations and for patient adherence monitoring along with online and offline mobile technology as a means of preventing relapse and the subsequent development of drug resistance, even outside pandemics.
2. Simultaneous TB screening or testing of suspected COVID-19 patients presenting with a cough.
3. Incorporating technological advances in COVID-19 management into TB control programs.
4. Highlighting the importance of staff within TB services to prevent them from being transferred to COVID-19 response services especially when new VoC are identified and are causing panic. Moreover, integrating epidemiological and genomic surveillance is essential for developing a comprehensive public health response strategy that is tailored to local needs [19].
5. Intensifying public education about TB especially in high-risk areas in order to prevent it from getting overshadowed by COVID-19.
6. Adopting a multi-sectoral integrated approach which addresses the social determinants of TB and improve data collection on the financial hardships incurred by TB patients and their families, to inform the development of supportive and targeted social programs.
7. Scaling-up surveillance and monitoring systems which would benefit both diseases as well as any future outbreak with an epidemic potential.
8. Advocating for unified public policies and inter-sectoral collaborations in order to properly respond to health emergencies such as COVID-19 in a timely manner, while simultaneously preserving the hard-won gains in the management of long-standing health problems such as TB.
9. Addressing social determinants such as improved nutrition, housing conditions, social protection and access to healthcare in order to lessen the impact of both transmissible infections as well as future ones.
10. Convening TB civil society partners in order to standardize TB prevention and control approaches, reduce fragmentation and align this effort with the national plan for health “Santé 2025”.

## Conclusion

As the article discusses the current pain points, situation and damage caused during COVID-19 to the TB affected people, a conclusion is made addressing the most pressing recommendations for further change. The article brings to light the unspoken challenges and hurdles faced by the people of Morocco. Creating optimal health standards and public policies is the ultimate of goal of combined efforts of researchers, program coordinators and governments on the whole. Further Global Burden of Disease analysis in association with global health policy-makers may pave a way forward to strengthen the current situation of Morocco.

## Data Availability

Not applicable.

## Abbreviations

COVID-19: Coronavirus disease 2019
TB: Tuberculosis
HIV: Human immunodeficiency virus
DOTS: Directly observed treatment short-course
NTP: National Tuberculosis Program
WHO: World Health Organization
MoH: Ministry of Health
MDR-TB: Multi-drug resistant tuberculosis
XDR-TB: Extensive drug-resistant tuberculosis
RR: Rifampicin resistance
